# Effects of Acupuncture on Leucopenia, Neutropenia, NK, and B Cells in Cancer Patients: A Randomized Pilot Study

**DOI:** 10.1155/2014/217397

**Published:** 2014-07-24

**Authors:** Irene Pais, Nuno Correia, Isabel Pimentel, Maria José Teles, Esmeralda Neves, Júlia Vasconcelos, Judite Guimarães, Nancy Azevedo, António Moreira Pinto, Jorge Machado, Thomas Efferth, Henry J. Greten

**Affiliations:** ^1^Instituto Ciências Biomédicas Abel Salazar, Universidade do Porto, Rua de Jorge Viterbo Ferreira No. 228, 4050-313 Porto, Portugal; ^2^Service of Emergency, Internal Medicine, Hospital Center of S. João, Oporto, Portugal; ^3^Service of Oncology, Hospital Center of S. João, Oporto, Portugal; ^4^Service of Clinical Pathology, Hospital Center of S. João, Oporto, Portugal; ^5^ISPUP-EPI Unit, Institute of Public Health, University of Oporto, Portugal; ^6^Service of Clinical Pathology, Porto Hospital Center, Oporto, Portugal; ^7^Department of Oncology, Hospital Center of Vila Nova de Gaia/Espinho, Portugal; ^8^Oporto Biomechanics Laboratory, Portugal; ^9^Department of Pharmaceutical Biology, Institute of Pharmacy and Biochemistry, Johannes Gutenberg University, Germany; ^10^Heidelberg School of Chinese Medicine, Germany

## Abstract

Chemotherapy is one of most significant therapeutic approaches to cancer. Immune system functional state is considered a major prognostic and predictive impact on the success of chemotherapy and it has an important role on patients' psychoemotional state and quality of life. In Chinese medicine, chemotherapy is understood as “toxic cold” that may induce a progressive hypofunctional state of immune system, thus compromising the fast recovery of immunity during chemotherapy. In this study, we performed a standardized acupuncture and moxibustion protocol to enhance immunity in cancer patients undergoing chemotherapy and to assess if the improvement of immunity status correlates with a better psychoemotional state and quality of life.

## 1. Introduction

Colorectal cancer (CRC) is one of the most common cancers and a major cause of death due to cancer worldwide. The functional state of the host immune system has a major prognostic and predictive impact on the fate of cancer patients treated with conventional or targeted chemotherapies [1].

According to the immunoediting theory [2], cancer cells and immune cells reciprocally modulate each other and the two possible outcomes are either the elimination or escape of tumour cells. NK cells are considered to represent a* first line of defence against the metastatic spread of tumour cells*. This idea is supported by the report of an association between the decreased activity or low numbers of circulating NK cells with progression of cancers and correlation between an absolute decrease in the activity of the NK cells and an absolute decrease in the lytic potential of these cells [3]. As effector members of the innate immunity, NK cells play a major role in anti-infection activity and tumour surveillance. NK cells can directly kill target cells to which they are capable of adhering within 1 to 4 hours without prior activation, priming or assistance by cytokines. NK cells have been recognized as major producers of cytokines in many physiological and pathological conditions, such as interferon *γ* (IFN*γ*), tumour necrosis factor (TNF*α*), and interleukin-10 (IL-10), as well as growth factors such as granulocyte macrophage colony-stimulating factor (GM-CSF), granulocyte colony-stimulating factor (G-CSF), and IL-3. NK cells also secrete several chemokines, which are vital for their colocalization with other hematopoietic cells such as dendritic cells (DC) in areas of inflammation.

Recent studies [4] provide the notion that tumour-induced alterations of activating NK cell receptor expression may hamper immune surveillance and promote tumour progression and reveal that NK cell activity is reduced in patients with metastatic CRC, pointing out to NK cells as a first line of defence against metastasis.

According to psychoneuroimmunology theory, psychological and emotional stress induces several alterations in diverse biological responses. The activation of the* hypothalamic-pituitary-adrenal axis* (HPA) and the* sympathetic nervous system *(SNS) may generate a change in the immune cell traffics and promotes inflammation via multiple neuroendocrine and immune pathways. Higher stress levels were associated with poorer immune responses (low NK cell activity) and the stress reduction with improvement of immune responses, in women with breast cancer [5].

Humoral and, especially, cellular immune functions have been reported as being boosted by acupuncture and moxibustion in cancer patients with significant increases in several T lymphocyte subsets. Immune system modulation has been noted also for various other conditions, such as asthma and autoimmune and inflammatory diseases [6, 7].

A review on the ascribed immunomodulation of acupuncture concludes that acupuncture treatment appears to be able to modulate immunosuppressed or immunoactivated conditions through different mechanisms, including macrophages, neutrophils, NK cells and lymphocytes stimulation, immunoglobulin production, and complement system activation [8]. In fact, two possible implicated mechanisms were previously reported: NK-related gene expression in the spleen [9] and the sympathetic nervous system [10]. Another study revealed that acupuncture enhances the NK cells activity and modulates the balance between Th1 and Th2 [11].

We aimed to evaluate the effect of acupuncture on the immune system (namely, on WBC, ANC, lymphocytes, and NK cells activity) on CRC patients and assess if the ascribed immunomodulatory effects of acupuncture have implications on patients' psychoemotional state and quality of life.

## 2. Patients and Methods

### 2.1. Selection of Patients

This study was approved by the Ethics Committee of S. João and Vila Nova de Gaia/Espinho Hospital Centers, Oporto, Portugal. Written informed consent was obtained from all patients before study enrollment. Patients were eligible for inclusion as follows: recently diagnosed or recurrent colorectal cancer, regardless of stage, receiving chemotherapy, no regular use of acupuncture within 120 days prior to enrollment, ability to give informed consent, and >18 years of age. Exclusion criteria were the following: (a) absolute neutrophil count (ANC) less than 500/*μ*L, (b) platelet count less than 25,000/*μ*L, (c) altered mental state, (d) clinically significant cardiac arrhythmias, and (e) other unstable medical conditions.

### 2.2. Study Design

**Figure 1:**
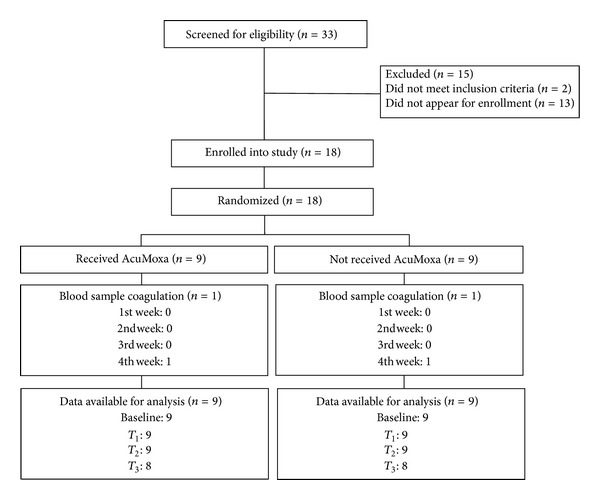
CONSORT flow of participants through the study.

All patients enrolled were evaluated at baseline. Patients were randomized into one of two groups: active acupuncture (AcuMoxa) or nonacupuncture (control group). All of the study patients were blinded to randomization assignments (Figure 1).

**Figure 2:**
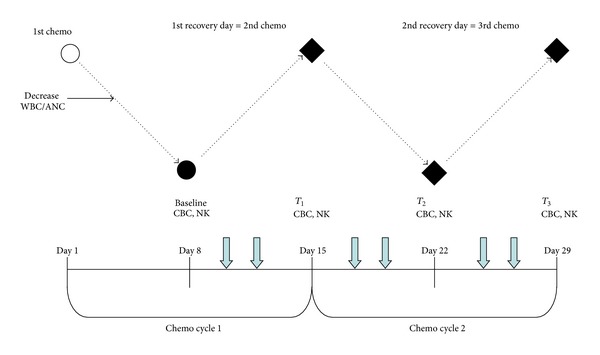
Study flow chart. Black solid dots: time points of outcome measurements. Open circle: first chemotherapy day. Black diamonds: the primary endpoints of the study. Dashed lines: the expected changes, during chemotherapy, of white blood cells (WBC) and absolute neutrophil counts (ANC). Short, blue down arrows: acupuncture treatments. CBC, complete blood counts; NK, NK cells and subsets.

Patients in the experimental group received 6 sessions of acupuncture, twice a week, beginning one week prior to cycle of chemotherapy and ending at the beginning of the following cycle of chemotherapy (Figure 2). At the end of the intervention, patients in the active arm continued their remaining chemotherapy cycles without any acupuncture treatment. Patients in the control arm were offered the active acupuncture protocol immediately after they completed the four weeks of blood sampling as a courtesy.

Based on previous trials [12, 13], blood samples were collected at baseline (preintervention) 7 days before chemotherapy and then every 7 days during four weeks. Blood samples of both groups, experimental and control, were taken under the same conditions.

### 2.3. Acupuncture Protocol

All acupuncture treatments were performed at S. João and Vila Nova de Gaia/Espinho Hospital Centres, Porto, Portugal. The acupuncture treatments were administered only by one acupuncturist (the main researcher; master degree on TCM by the University of Porto). A standardized acupuncture protocol was developed based on the Heidelberg model of Chinese medicine (CM), in which CM is explained as a comprehensive model of system biology based on a technical understanding of the regulatory core termini of CM, such as Yin, Yang, the phases, and the Shan Hang Lung theory [14–17].

The experimental group (AcuMoxa): acupuncture points and their anatomical locations were as follows: lower extremity (LV3, ST36, SP3, and GB39) and upper extremity (LI4, PC5, TB5, and LU7), and disposable acupuncture needles with a size of 36 G, 0.20 × 25 mm (Tewa). The depth of needling was at approximately 10 mm. The* de qi* sensation was required [18]. Smokeless moxibustion treatment was used in the following points: SI6, TB5, ST32, and CV6; 2 minutes per point. Each session had a duration of 45 minutes.

### 2.4. Clinical and Laboratory Evaluation

Complete blood counts were collected at baseline and then once every 7 days at 3 time points during the study period (Figure 2). The timing for collecting blood samples was based on previous trials [12, 13].

Lymphocyte populations T, B, and NK were analysed by flow cytometry (Coulter, EPICS XL-MCL flow cytometer) with a combination of monoclonal antibodies anti-CD3-FITC/(CD56 + CD16)-PE (Immunotech).

Anxiety and depression scores as well as patients quality of life were assessed through Hospital anxiety and depression scale (HADS) and EORTC-QoL CR-29 questionnaires, respectively, in the beginning and at the end of the study for both groups.

### 2.5. Statistical Analysis

This study was designed to provide preliminary data about its feasibility and analysis to support a subsequent large-scale, fully powered study to evaluate the effects of acupuncture on NK cells in patients with CRC.

Differences between groups were assessed through Mann-Whitney *U* test. Intragroup analyses were assessed by Friedman test. In order to explore variables correlations, the Pearson correlation analyses was performed. Missing data were handled based on available data approach. SPSS for Windows was used for statistical computation. Results yielding a *P* value < 0.05, with alpha = 0.05 and C.I. level = 95, were considered statistically significant.

## 3. Results

### 3.1. Baseline Sample Characterization

At baseline, patients in both groups shared similar demographic and clinical characteristics (Table 1). The majority of patients (88.9%) have a colon cancer, with Stage II (16.7%) and Stage III (55.6%); 66.7% of the patients were submitted to the FOLFOX and 16.7% to XELOX chemotherapy regimens.

Comparison of blood analyses at baseline showed no statistical differences among groups (Table 1).

A total of   18 (100%) patients completed the questionnaires of QOL-CR29 and HADS at the beginning and at the end of the study.

Comparison of QOL and anxiety and depression scores at baseline did not reveal any statistical differences among groups (Table 1); however the experimental group revealed high scores of depression.

### 3.2. Effect of Acupuncture on WBC and ANC

**Figure 3:**
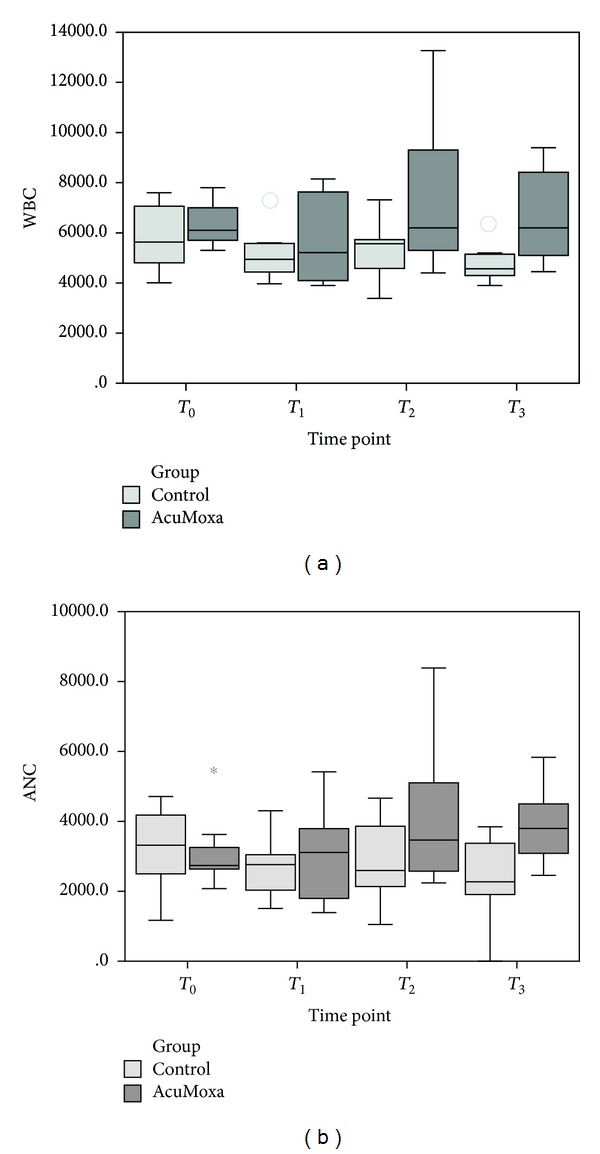
WBC and ANC. Comparison between groups.

The effects of acupuncture treatment on WBC counts and ANC in study patients are shown in Figure 3. Comparison analyses between the two groups showed that AcuMoxa group had statistically significant higher values on WBC (*P* = 0.036) as well as on ANC (*P* = 0.046) at time point *T*_3_.

Within group analyses showed that the changes in each group across time were significant: in the control group diminishing levels of WBC were seen (*χ* ^2^ = 7.8, *P* = 0.046, *n* = 8); on AcuMoxa group (*n* = 9), there was an increasing level of these parameters overtime (*χ* ^2^ = 11.011, *P* = 0.012; *χ* ^2^ = 11.966, *P* = 0.012, resp.) as well as the ANC (*P* = 0.007).

### 3.3. Effect of Acupuncture on Lymphocyte Populations

**Figure 4:**
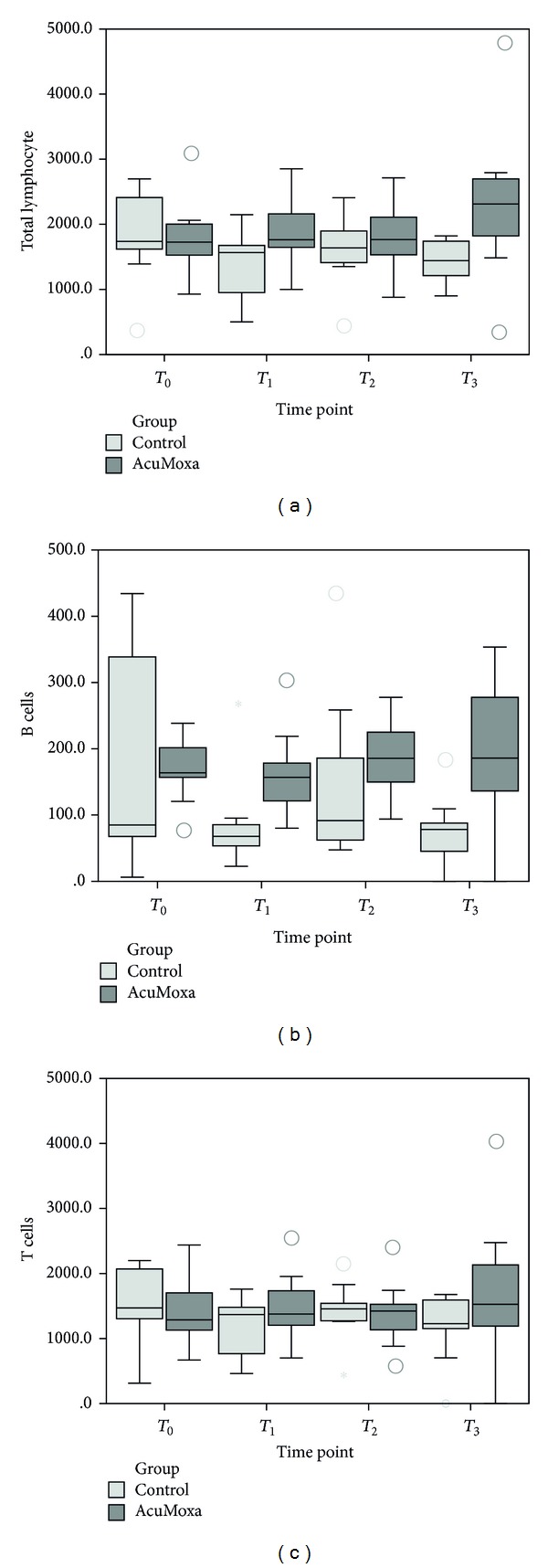
Total lymphocytes and T and B cells. Comparison between groups.

Although differences on total lymphocyte populations among control and experimental group were identified only after 6 sessions of AcuMoxa treatment (*P* = 0.021), higher values of B cells at time point *T*_1_ (*P* = 0.009) and *T*_3_ (*P* = 0.002) were seen in the AcuMoxa group. With respect to T cells, no statistical differences among groups were found (Figure 4).

Total lymphocytes as well as B and T cells intragroup analyses did not reveal differences across time.

### 3.4. Effect of Acupuncture on NK Cells

**Figure 5:**
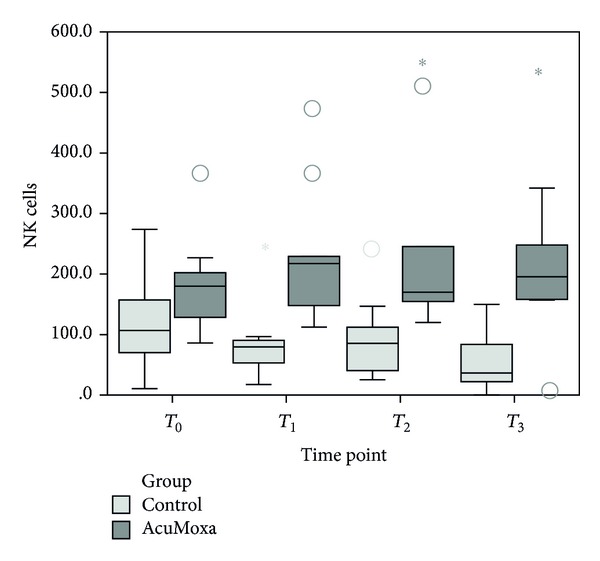
Total NK cells counts. Comparison between groups.

Comparison between groups showed significant higher values of NK cells on the AcuMoxa group from time point *T*_1_ until time point *T*_3_ (*P* = 0.002, *P* = 0.003 and *P* = 0.000, resp.) (Figure 5).

Within group analyses revealed significant increases on NK cells across time (*χ* ^2^ = 7.8, *P* = 0.046, *n* = 8), while the control group showed a significant decrease of NK cells (*χ* ^2^ = 7.65, *P* = 0.049, *n* = 8).

### 3.5. Effect of Acupuncture on Anxiety and Depression Levels

**Figure 6:**
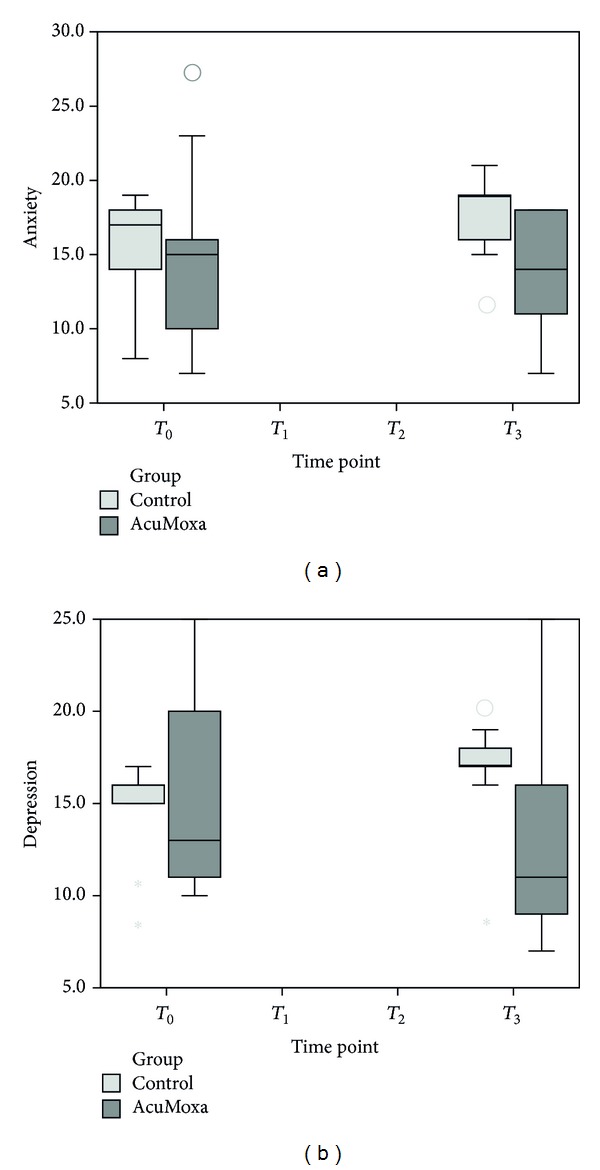
HADS scores. Differences among groups, at beginning (*T*_0_) and at the end (*T*_3_) of the study.

The comparison between the two groups did not reveal significant differences on anxiety (0.050) and depression levels (0.094) (Figure 6). However, when analyzing each group, it was shown that the experimental group had a significant decrease depression mean (*P* = 0.027), whereas in the control group the anxiety (*P* = 0.031) and depression (*P* = 0.027) levels increased significantly. In addition, exploratory analyses on a possible correlation between NK cells and depression and anxiety levels did not reveal any significant correlation, despite the observation of a decrease on anxiety and depression levels along with an increase of NK cells.

### 3.6. Effect of Acupuncture on Patients QOL

**Figure 7:**
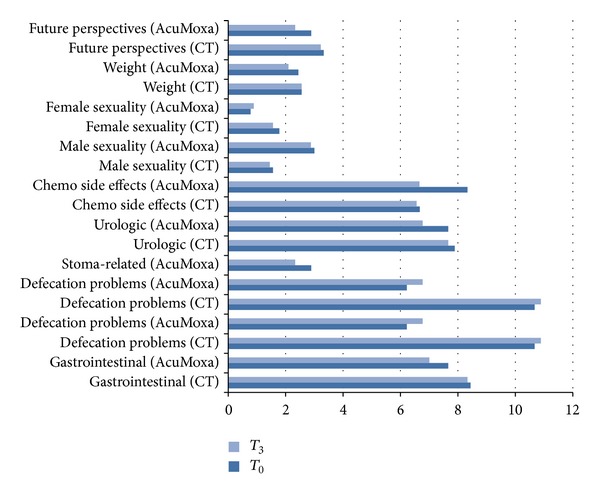
Symptoms related to QOL questionnaires. Differences among groups at the beginning (*T*_0_) and at the end (*T*_3_) of the study. CT, control group; AcuMoxa, experimental group.

With respect to the analyses of the different items of QOL questionnaires, no significant differences were identified between the control and experimental groups (Figure 7). However, data collected from QOL questionnaires showed a tendency of reduction of several symptoms such as gastrointestinal symptoms, urological symptoms, stoma-related symptoms, male sexual dysfunction, and chemotherapy side effects (Figure 5).

**Figure 8:**
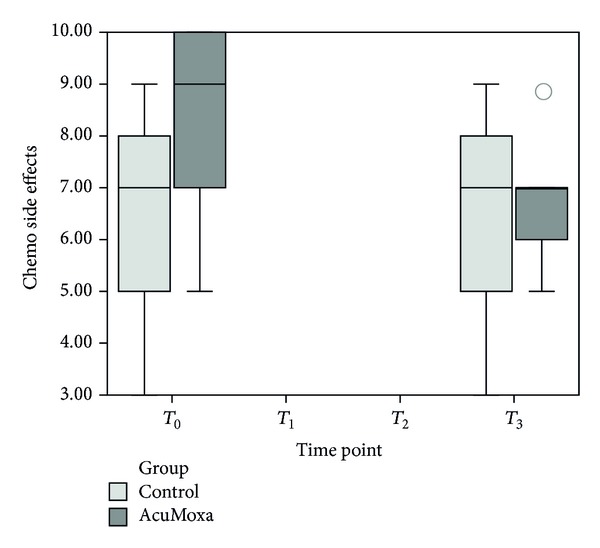
Chemotherapy side effects. Intragroup analyses show significant decrease of chemotherapy side effects among AcuMoxa group.

Intragroup variability analyses showed a significant decrease of chemotherapy side effects (Figure 8) on the experimental group (*P* = 0.034).

## 4. Discussion

The host immune system functional state has a major prognostic and predictive impact on the outcome of cancer patients treated with conventional or targeted chemotherapies [1].

Several authors revealed that acupuncture modulates NK cell number and function in diverse clinical situations such as in women with severe anxiety [19], in pain syndromes [20], and in healthy volunteers [21].

Studies regarding the effect of acupuncture and moxibustion on CRC patients are scarce. In fact, only two eastern studies have addressed modulation of NK cells activity in CRC patients [22, 23].

To the best of our knowledge, this research protocol is the first study on acupuncture for cancer patients conducted in the Portuguese National Health System and is the first controlled clinical trial on the West that addressed acupuncture and moxibustion NK cells modulation, its implications on psychoemotional state, and on the QOL of CRC patients.

Although our data must be carefully interpreted due to methodological limitations, some conclusions may be pointed out.

Firstly, we observed (1) a reduction on anxiety and depression and (2) consistent positive trends on the levels of WBC, ANC, and B and NK cells in the AcuMoxa group versus the control group. The increase on WBC and ANC resulted in approximately a 1.5x reduction in leukopenia and neutropenia rates. The acupuncture group showed a twofold increase in NK cells rate compared to the control group. These preliminary results indicate an immunomodulatory effect of acupuncture in CRC patients undergoing chemotherapy. Acupuncture stimulation may yield a myeloprotective effect as suggested by Lu et al. on a study on electroacupuncture plus TDP infrared lamp effect in gynaecologic malignancies. Despite the differences in the selection of acupoints and type of acupuncture, WBC and ANC levels were similar to those obtained by Lu et al.

Secondly, our preliminary results show that acupuncture benefits the emotional status by decreasing anxiety and depression levels. This effect may contribute to improvement on NK cells activity. As reported recently on a study on women with breast cancer [24], the emotional state influences NK cells numbers and activity.

Thirdly, with respect to quality of life, our study did not reveal significant differences between the two groups. However, we observed a tendency for decreasing certain symptoms on the AcuMoxa group, such as gastrointestinal and urological symptoms and chemotherapy side effects as well as the improvement of sexual function in men. This is probably due to the short period of treatment. In addition, intragroup analyses reveal a significant decrease of chemotherapy side effects on the AcuMoxa group indicating an overtime protective role of acupuncture for CRC patients during chemotherapy.

Fourthly, no acupuncture and moxibustion-related adverse events were observed. Globally, these preliminary results indicate that our AcuMoxa protocol is feasible and safe for CRC patients undergoing chemotherapy.

What may be the physiological explanation for the observed results of AcuMoxa stimulation?

It is generally accepted that acupuncture induces an increase on the release of *β*-endorphin [5, 23] via the stimulation of the HPA axis. *β*-Endorphins consequently influence immune cells by binding to opioid receptors on the surface of the cells, namely, on NK cells [25] promoting the expression of cytotoxic molecules and the production of IFN*γ*. In turn, IFN*γ* would further increase the expression of NK cells receptors and cytokine secretion by other immune cells, thereby amplifying anticancer immune functions.

The HPA axis and SNS are generally activated in cancer, resulting in high levels of catecholamine and glucocorticoids, which augments the sympathetic outflow and decreases NK activity in the periphery [25].

Therefore, we may hypothesize that acupuncture, by acting on the SNS and the HPA axis, may reduce the levels of catecholamines and consequently attenuate their suppressive effects on NK cells.

There are limitations in our preliminary study to be considered.

1. Although the patients were randomly allocated in each group, there is the possibility that results may have occurred by chance due to the small sample size. A larger study based on this protocol is required to precisely evaluate the effects of acupuncture on CRC patients during chemotherapy and to explore the relation of the cancer severity and different types of chemotherapy on the possible acupuncture immunomodulatory effect. Nevertheless, the small sample of patients allowed assessing the trial feasibility and preliminary data on efficacy.
2. Another limitation of our study was the heterogeneity on chemotherapy protocols which may have influenced the hypothesized AcuMoxa effects and results.
3. Finally, the short duration of the study did not allow obtaining more precise data regarding the impact of acupuncture on patients QOL and prognosis.

## 5. Conclusions

Our pilot study suggests that acupuncture and moxibustion may (1) stimulate anticancer immunity, (2) promote a myeloprotective effect, (3) improve the psychoemotional status and quality of life, and (4) minimize chemotherapy side effects.

This study protocol proved to be feasible and safe for CRC patients.

A larger and long-term acupuncture trial is needed to clarify acupuncture's immunomodulatory effects in CRC. If this effect is ultimately established, then this treatment may serve as a possible complementary therapy for CRC treatment and possibly contribute to improving patients' prognosis and quality of life.

## Figures and Tables

**Table 1:** Baseline comparison between groups: sociodemographic and clinical characteristics; blood analyses and QOL-CR 29 and HADS scores.

Characteristics	Experimental group (*n* = 9)	Control group (*n* = 9)
Age		
Mean; range	62.2; 38–74	56.6; 43–74
Cancer type		
Colon	8 (88.9%)	9 (100%)
Rectum	1 (11.1%)	0 (0%)
Tumor staging		
II	1 (11.1%)	2 (22.2%)
III	7 (77.8)	3 (33.3%)
IV	1 (11.1%)	4 (44.4%)
Type of chemotherapy		
FOLFOX	6 (66.7%)	6 (66.7%)
XELOX	1 (11.1%)	2 (22.2%)
FOLFIRI	1 (11.1%)	0
Capecitabine	1 (11.1%)	1 (11.1%)
Blood analyses (*P* value)	**(mean cells/** ***μ*** **L; s.d.)**	**(mean cells/** ***μ*** **L; s.d.)**
WBC (*P* = 0.400)	6367.78; 897.55	5915.56; 1288.59
ANC (*P* = 0.864)	3128.78; 996.25	3219; 1199.07
Total lymphocyte (*P* = 0.736)	1734.59; 586.98	1842.28; 735.77
B cells (*P* = 0.978)	170.88; 51.59	169.25; 168.91
T cells (*P* = 0.542)	1378.95; 540.62	1548.76; 613.11
NK cells (*P* = 0.146)	184.52; 81.07	123.44; 88.46
QOL-Cr 29 and HADS scores (*P* value)	**(mean; s.d.)**	**(mean; s.d.)**
Anxiety (*P* = 0.722)	14.78; 6.45	15.67; 3.53
Depression (*P* = 0.432)	16.11; 5.84	14.33; 3.12
Urological symptoms (*P* = 0.632)	7.67; 1.00	7.89; 0.93
Gastrointestinal symptom (*P* = 0.609)	7.67; 2.74	8.44; 3.54
Defecation symptoms (*P* = 0.78)	6.22; 6.05	10.67; 3.67
Stoma-related symptoms (*P* = 0.66)	2.89; 4.40	—^a^
Chemo side effects (*P* = 0.96)	8.33; 1.93	6.67; 2.06
Male sexual function (*P* = 0.239)	3.00; 2.59	1.56; 2.40
Female sexual function (*P* = 0.148)	0.78; 1.20	1.78; 1.56
Weight concerns (*P* = 0.857)	2.44; 1.24	2.56; 1.33
Body image (*P* = 0.819)	4.56; 1.42	4.78; 2.49
Future concerns (*P* = 0.398)	2.89; 1.36	3.33; 0.71

(S.d., standard deviation; *P* < 0.05 considered statistically significant; —^a^, no patients with stoma bag on the control group.)
